# Metal-Assisted Complexation of Fluorogenic Dyes by Cucurbit[7]uril and Cucurbit[8]uril: A DFT Evaluation of the Key Factors Governing the Host–Guest Recognition

**DOI:** 10.3390/molecules28041540

**Published:** 2023-02-05

**Authors:** Nikoleta Kircheva, Stefan Dobrev, Lyubima Dasheva, Valya Nikolova, Silvia Angelova, Todor Dudev

**Affiliations:** 1 Institute of Optical Materials and Technologies “Acad. J. Malinowski”, Bulgarian Academy of Sciences, 1113 Sofia, Bulgaria; 2 Faculty of Chemistry and Pharmacy, Sofia University “St. Kliment Ohridski”, 1164 Sofia, Bulgaria

**Keywords:** cucurbituril, thiazole orange, neutral red, thioflavin T, complexation

## Abstract

With the emergence of host-guest systems, a novel branch of complexation chemistry has found wide application in industries such as food, pharmacy, medicine, environmental protection and cosmetics. Along with the extensively studied cyclodextrins and calixarenes, the innovative cucurbiturils (CB) have enjoyed increased popularity among the scientific community as they possess even better qualities as cavitands as compared to the former molecules. Moreover, their complexation abilities could further be enhanced with the assistance of metal cations, which can interestingly exert a dual effect on the complexation process: either by competitively binding to the host entity or cooperatively associating with the CB@guest structures. In our previous work, two metal species (Mg^2+^ and Ga^3+^) have been found to bind to CB molecules in the strongest fashion upon the formation of host–guest complexes. The current study focuses on their role in the complex formation with three dye molecules: thiazole orange, neutral red, and thioflavin T. Various key factors influencing the process have been recognized, such as pH and the dielectric constant of the medium, the cavity size of the host, M^n+^ charge, and the presence/absence of hydration shell around the metal cation. A well-calibrated DFT methodology, solidly based and validated and presented in the literature experimental data, is applied. The obtained results shed new light on several aspects of the cucurbituril complexation chemistry.

## 1. Introduction

Supramolecular chemistry is a field of enormous potential finding application in both scientific research and industry. A great variety of cavitands have been recognized and extensively studied, among which are cyclodextrins [1,2], calixarenes [3], and crown-ethers [4]. A promising entrant to this class, however, is the cucurbituril family [5,6,7,8,9]. Cucurbit[n]urils, CB[n]s, where n equals 5 to 10 (without 9) and 13 to 15, are obtained through a relatively simple reaction of the condensation of glycoluril with formaldehyde in strongly acidic media, thus resulting in the formation of a macrocycle containing *n* glycoluril units connected through two methylene bridges on each side of the segments [10,11,12,13]. As a consequence, CBs possess unique structural properties: they are rigid, with a highly symmetrical form resembling that of a pumpkin (hence the name), where the two identical carbonyl-laced portals enclose a hydrophobic inner cavity with low polarity and polarizability [14]. Due to their inflexible structure, all cucurbiturils have the same height (9.1 Å) and differ only in portal size and cavity diameter (and therefore in cavity volume) [10,12]. Their thermal stability (above 370 °C) is also remarkable [8]. The partially negatively charged C=O moieties allow the cavitands to incorporate cationic guests through ion-dipole interactions on the outer surface, while the inner space encapsulates the guest molecules through noncovalent bonding. By the size of the cavity, CB[6], CB[7], and CB[8] are analogs of α-, β-, and γ-cyclodextrin, respectively. Nonetheless, the resulting CB–guest complexes possess larger stability constants, which can even be several orders of magnitude higher for guests bearing a positive charge [15,16,17,18]. All these factors provide a solid premise for their implementation as on–off switches [19,20] in targeted drug delivery [21,22,23,24,25], enzymatic assays [26], and as supramolecular architectures–bracelets and necklaces [27,28,29]. Due to their low toxicity and high biocompatibility, CBs and their resulting complexes are utilized in various branches of pharmacy and medicine [30,31,32].

An intriguing aspect of cucurbiturils’s chemistry concerns their ability to incorporate fluorescent dyes with medicinal application, thus shifting the corresponding p*K_a_* values of the host species [33,34,35,36,37,38,39]. As a consequence, the fluorescence intensity, brightness and photostability are increased, and the guest molecule is protected towards fluorescence quenchers and aggregation. Moreover, the solubility in polar environments (especially when the host is CB[7]) is enhanced. Numerous studies have explored the complexation abilities of CBs with different guest dyes. The interactions between fluorescent dyes and water-soluble macrocyclic hosts have been reviewed in detail with an emphasis on the fluorescence of the dyes upon complexation [40]. For the purposes of the current research, however, the following three dyes (in their neutral and/or cationic forms) were chosen as their complexes with CBs possess curious structural and physicochemical properties: thioflavin T (TfT), thiazole orange (TO), and neutral red (NR). Thioflavin T finds a broad application in medicinal chemistry as a diagnostic dye since its fluorescence enhances dramatically (more than 1000 times) upon binding tightly to amyloid fibrils—a hallmark for neurodegenerative diseases such as Alzheimer’s and Parkinson’s [33,41,42]. A significant reduction in the non-radiative processes is observed when the torsion angle between the benzothiazole and dimethylanilinium groups is restricted, thus favoring its radiative transition [43]. Thiazole orange, on the other hand, is a cyanine-based dye, which is non-fluorescent in water, but displays substantial enhancement in its quantum yield in a viscous solution or when interacting with nucleobases [37,44,45]. It is, therefore, applied as a fluorogenic sensor reporting on the viscosity of the surrounding medium or as an optical DNA/RNA detector [46,47]. Neutral red, as a third example, is a phenazine-based dye which finds diverse applications as a fluorescent probe and marker in biological systems due to its relatively non-toxic nature and p*K_a_* of 6.8, rendering it a reliable pH indicator near the physiologically most relevant region [48,49,50]. The corresponding structures of the dyes in their protonated form, along with their IUPAC names, are given in Table 1.

Herewith, we endeavor to continue our previous work [51,52] exploring key factors governing metal-assisted cucurbituril–guest complexation activity by applying reliable DFT/SMD methodology. Metal cations play a crucial role in host–guest recognition but are known to exert a dual effect by either competitively binding [53] or cooperatively associating [54] with the CB@dye structures. Hence, a systematic study based on the provided literature experimental data, but utilizing the powerful tools of the in silico approach, is highly required. The obtained results aim at giving a clear answer to the intriguing questions regarding the role of the metal cations in cucurbituril–dyes host–guest complexation processes at the molecular level.

## 2. Results

### 2.1. Reactions Modelled

In order to account for the diverse factors governing the host–guest recognition process, the following reactions were modelled:

CB[7/8] + dye^0/+^ → CB[7/8]@dye^0/+^ (R1)

CB[7/8]@dye^+^ + M^n+^ → CB[7/8]@M^n+^ + dye^+^ (R2)

CB[7/8]@dye^+^ + M^n+^ → CB[7/8]@dye^+^@M^n+^ (R3)

Reaction (R1) illustrates the formation of a CB[7/8]@dye complex, where the two most popular representatives of the cucurbituril family are modelled, thus elucidating the effect of the cavity volume. The pH of the surrounding medium is additionally assessed by modelling both the neutral and positively charged forms of the dye, neutral red. Furthermore, the effect of high-energy water molecules present in the host cavity is considered when a CB[7]@8W is taken into account as an initial structure in the reaction, which loses the water cluster during the process of the CB[7]@dye formation. CB[7] is reported to encapsulate eight high-energy water molecules, according to Ref. [7]. Reactions (R2) and (R3) take into consideration the effect of the metal cation and its charge, as the first example is a competitive binding (substitution), while the second one represents a cooperative association (addition). The presence/absence of a hydration shell around the metal cation was also taken into account.

**Figure 1:**
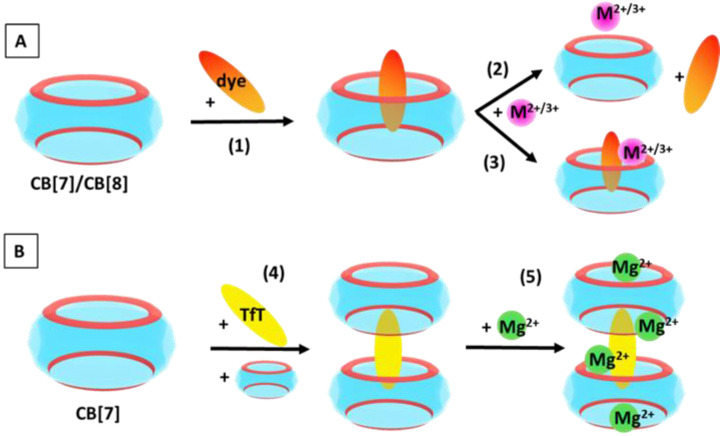
General scheme of the reactions modelled in the current study. (**A**) Formation of host:guest complexes: CB[7/8]@dye according to (1); CB[7/8]@M^2+/3+^ (competitive binding of metal cations in ^2+/3+^ oxidation state) according to (2); ternary CB[7/8]@dye@M^2+/3+^ structures (cooperative association of metal cations in ^2+/3+^ oxidation state) according to (3). (**B**) Stepwise formation of supramolecular architectures: 2:1 CB[7]:TfT^+^ complexation in accordance to (4) and further addition of Mg^2+^ cations as given in (5).

The formation of a ternary complex with the assistance of metal (Mg^2+^) cations between CB[7] and thioflavin T is also modelled according to:

$$2*\mathrm{CB}[7]\;+\;{\mathrm{TfT}}^{+}\overset{\left(R4\right)}{\to }\;\mathrm{CB}[7]@{\mathrm{TfT}}^{+}@\mathrm{CB}[7]\;+\;4{\mathrm{Mg}}^{2+}\overset{\left(R5\right)}{\to }\;2{\mathrm{Mg}}^{2+}@\mathrm{CB}[7]@{\mathrm{TfT}}^{+}@\mathrm{CB}\;[7]@2{\mathrm{Mg}}^{2+}$$

where reaction (R4) illustrates the formation of a 2:1 host:guest complex, while in reaction (R5), two metal cations act as “lids” at the two outer portals of the ternary structure, and two additional magnesium cations are placed amid the inner transitional rims. The summarized reactions modelled in the present study are depicted in Figure 1.

### 2.2. 1:1. CB:Dye Complexes

**Figure 2:**
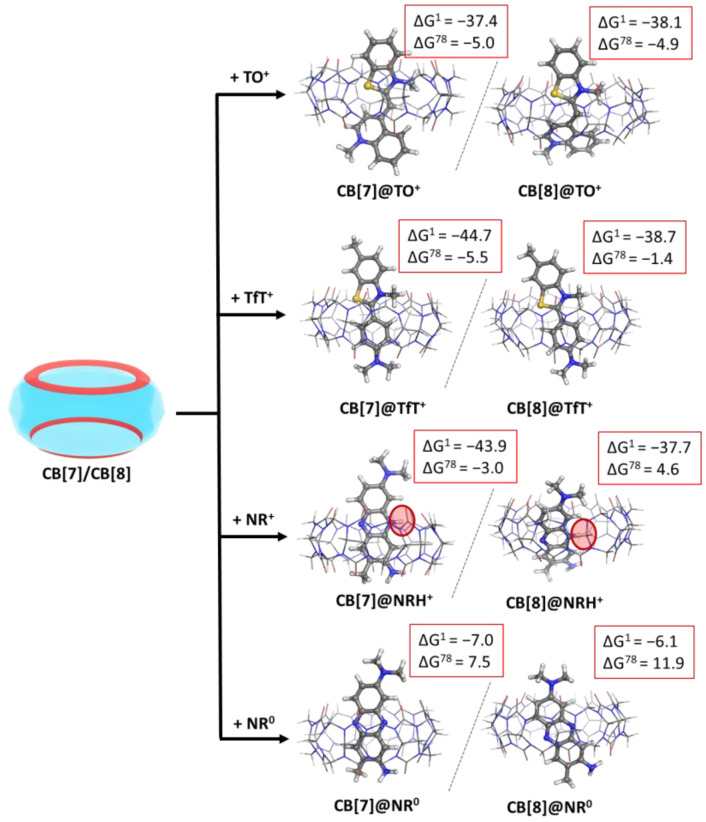
M062X/6−31G(d,p) optimized structures of the CB[7/8]@dye complexes in the gas phase and the corresponding ∆G^ε^ values in kcal mol^−1^ for their formation in the gas phase (ε = 1), and water (ε = 78) at the M062X/6−31+G(d,p)//M062X/6−31G(d,p) level of theory.

As a first step in the current study, we modelled the reactions of complexation between CB[7]/CB[8] and the three dye molecules according to reaction (R1). In the case of neutral red, both neutral and cationic forms were taken into account. The summarized results, along with the optimized CB@dye structures, are given in the following Figure 2 and Table S2 (see Supplymentary Materials).

### 2.3. Effect of pH of the Medium (Neutral vs. Positively Charged Dye)

The obtained results indicate that both CB[7] and CB[8] readily bind the two forms of neutral red in the gas phase, as the calculated Gibbs energies of complex formation are negative: −43.9 vs. −7.0 (CB[7]@dye) and −37.7 vs. −6.1 kcal mol^−1^ (CB[8]@dye) for the NRH^+^ vs. NR^0^, respectively. Still, the much greater preference towards the cationic structure is obvious and undoubtedly expected, since the arising ion–dipole interactions between the carbonyl moieties at the two portals and the positively charged N-atom from the dye stabilize the complex. In a water environment, however, only the formation of the CB[7]@NRH^+^ is thermodynamically possible in accordance with the calculated ∆G^78^: −3.0 kcal mol^−1^ [15].

### 2.4. Effect of the Cavity Size

By modelling the binary CB@dye complexes with either CB[7] or CB[8], the effect of the cavity size was assessed. The provided data show evidence that both cucurbiturils readily encapsulate the protonated guest molecules in the gas phase and water solution, with a slight preference towards the smaller representative. This outcome is mainly due to the closer interaction between the charged moieties in the guest and the carbonyl-fringed rims in the host stemming from the decreased portal size. The only exception poses the formation of a CB[8]@NRH^+^ complex. The tilted position of the dye, which occurs due to a greater cavity size during the optimization process, should be attributed to this outcome.

### 2.5. Effect of High-Energy Water Molecules in the Host Cavity

**Figure 3:**
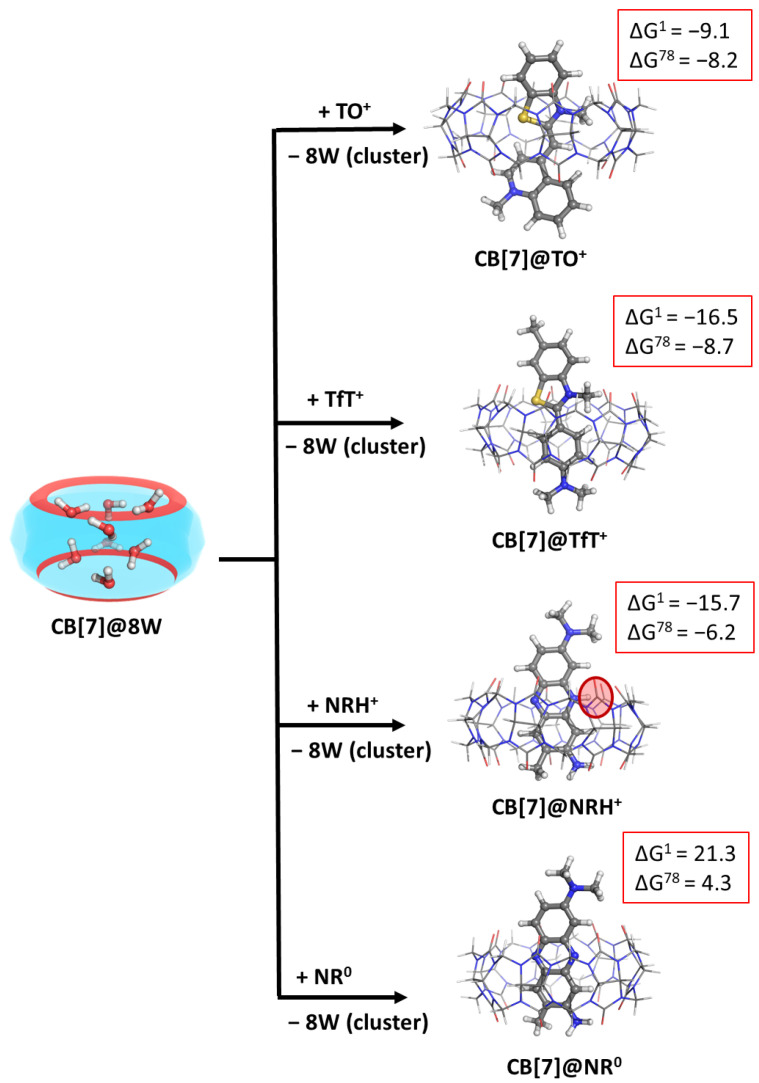
M062X/6−31G(d,p) optimized structures of the CB[7]@dye complexes in the gas phase and the corresponding ∆G^ε^ values in kcal mol^−1^ for their formation in the gas phase (ε = 1), and water (ε = 78) at the M062X/6−31+G(d,p)//M062X /6−31G(d,p) level of theory. The results are obtained by considering the cucurbituril with high-energy water molecules (denoted as CB[7]@8W) as an initial structure. The coming dye molecule/ion fully substitutes the water cluster from the host cavity.

The presence of the so-called high-energy water molecules in the cavity of the host may affect the outcome of the fluorogenic dye–CB complexation process. Thus, we modelled the previously observed reactions, but now the CB[7]@8W was considered as an initial structure, which loses its water cluster in order to form the corresponding CB[7]@dye complex. The calculated Gibbs energies of formation are presented in Figure 3 and Table S3.

The results imply that the present high-energy water molecules may have a significant role in the reaction: their replacement by a dye molecule from the host inner cavity leads to an energy gain of approximately 3.2 kcal mol^−1^ in an aqueous solution (compare results presented in Tables S1 and S2). Note that the effect of the water cluster brings the theoretical results near to the experimentally observed ones, as, for example, the reaction of the formation of a CB[7]@NRH^+^ has been estimated to be ≈ −7.9 kcal mol^−1^ (K_a_ = 6.10^5^ M^−1^), according to Ref. [15], which stays in good agreement with the calculated −6.2 kcal mol^−1^. Still, the aim of the conducted study is to observe trends and outline significant factors that contribute to the host–guest complexation process.

### 2.6. Effect of the Metal Cation

**Figure 4:**
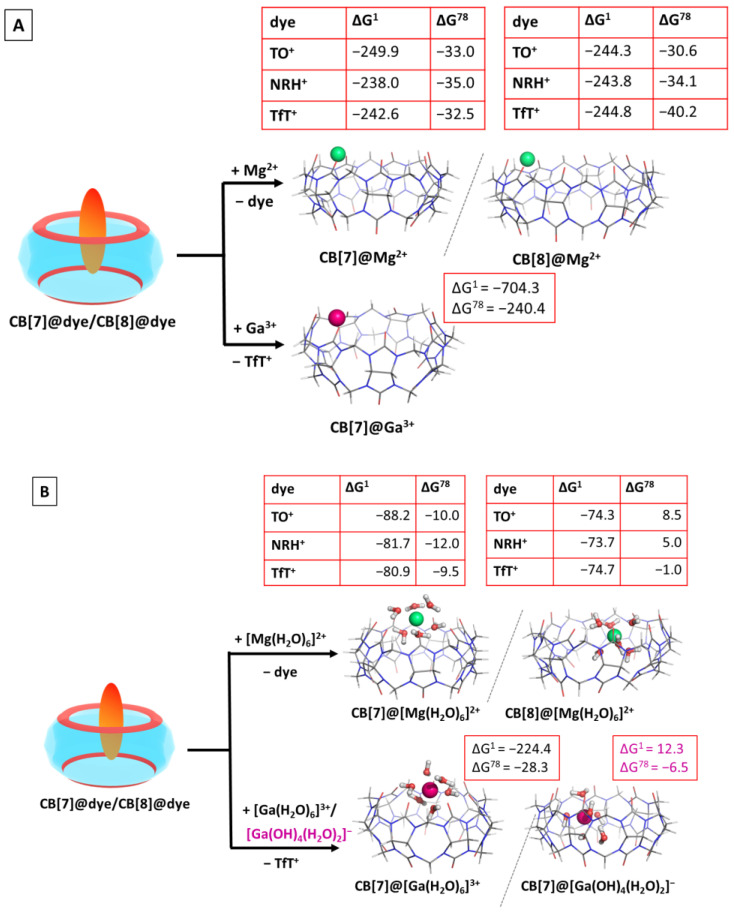
M062X/6−31G(d,p) optimized structures of the CB[7/8]@metal complexes in the gas phase and the corresponding ∆G^ε^ values in kcal mol^−1^ for their formation in the gas phase (ε = 1), and water (ε = 78) at the M062X/6−31+G(d,p)//M062X /6−31G(d,p) level of theory by substituting the dye ion from the host cavity. The results are obtained by considering the bare (**A**) and hydrated (**B**) metal cations. The hydration shell around gallium(III) is modelled as [Ga(H_2_O)_6_]^3+^/[Ga(OH)_4_(H_2_O)_2_]^−^ in correspondence with its pK_a_ values.

Our previous studies provided evidence that among biologically essential mono- and divalent metal cations, the magnesium ion binds to CBs in the strongest fashion [51], whereas the non-biogenic trivalent metal cations (e.g., gallium(III)) form even stronger complexes with the host cavitands [52]. In light of these findings, in the present research, we modelled Mg^2+^ and Ga^3+^ as competitors of the fluorogenic dyes. The obtained results are summarized in Table S4, whereas the optimized structures of the corresponding CB@M^n+^ binary complexes are depicted in Figure 4.

The series of reactions were thoroughly calculated for the magnesium cation. The calculated Gibbs energies show a clear trend of thermodynamically possible substitution in both the gas phase and aqueous medium, when the bare cation is considered. The ∆G^78^ values vary from −30.6 to −40.2 kcal mol^−1^ (for the substitution of CB[8]@TO and CB[8]NRH+, respectively). Adding a hydration shell around the metal cation changes the outcome. The substitution of the dyes in the CB[7] complexes is thermodynamically possible, as the corresponding Gibbs energies stay firmly on negative ground. Still, the corresponding ∆G^78^ decreases significantly in absolute value due to the lost intermediate ion-dipole interaction between the magnesium cation and the carbonyl moieties arising from the presence of shielding water molecules. Furthermore, the formation of a CB[8]@[Mg(H_2_O)_6_]^2+^ by competitively binding the host molecule seems possible only in the case of a substitution of NRH^+^ (∆G^78^ = –1 kcal mol^−1^), which is close to the error of the method. This outcome should be attributed to the cavity size and to the nature of the incoming [Mg(H_2_O)_6_]^2+^. In the CB[7]@[Mg(H_2_O)_6_]^2+^, all water molecules are engaged in hydrogen bonds with the carbonyl moieties from the host, whereas in the corresponding CB[8] complex, the H_2_O cluster is partially buried in the hydrophobic void, thus decreasing the number of intermolecular dipole–dipole interactions. Additionally, an empirical rule stipulates that a cucurbituril host exerts its best complexating abilities when the guest molecule “fills” approximately 55% of its cavity. By increasing the cavity size in CB[8], in comparison to CB[7], its interior is filled to a lesser extent by the incoming [Mg(H_2_O)_6_]^2+^ guest. These observed trends are in line with previous results [51] showing the greater preference of the metal cation toward a smaller host.

The effect of the abiogenic gallium cation was assessed upon substituting the dye from the CB[7]@TfT+ structure. Due to its higher charge of ^3+^, the bare gallium(III) exerts even better complexation abilities than Mg^2+^, seen by the extremely negative ∆G^1/ 78^ values, being −704.3 and −240.4 kcal mol^−1^, respectively. This result supports once again the thesis that the charge of the guest affects the outcome of the reaction to a great extent. When the hydrated cation is modelled as a reactant, two possible Ga^3+^ aqua complexes were considered due to gallium’s hydration constants (pK_a1_ = 3.09, pK_a2_ = 3.55, pK_a3_ = 4.4, and pK_a4_ = 6.05 [55]): [Ga(H_2_O)_6_]^3+^, present at pH ≈ 2, and [Ga(OH)_4_(H_2_O)_2_]^−^, existing at an ambient pH of 7. As it could be expected, the cationic hexa–aqua complex substitutes the dye much more easily from the CB[7] cavity as compared to its anionic counterpart, evidenced by the ∆G^78^ values: −28.3 vs. −6.5 kcal mol^−1^ for the formation of CB[7]@[Ga(H2O)6]3+, and CB[7]@[Ga(OH)_4_(H_2_O)_2_]^−^, correspondingly. Due to the ion-dipole repulsion between [Ga(OH)_4_(H_2_O)_2_]^−^ and the carbonyl groups from the cucurbituril along with the decreased number of hydrogen bonds, the resulting CB[7]@[Ga(OH)_4_(H_2_O)_2_]^−^ complex is not as stable as the CB[7]@[Ga(H_2_O)_6_]^3+^ one, resulting in a lower energy gain upon its formation.

### 2.7. Ternary Complexes

#### 1:1:1. CB@Dye@Metal Cation

**Figure 5:**
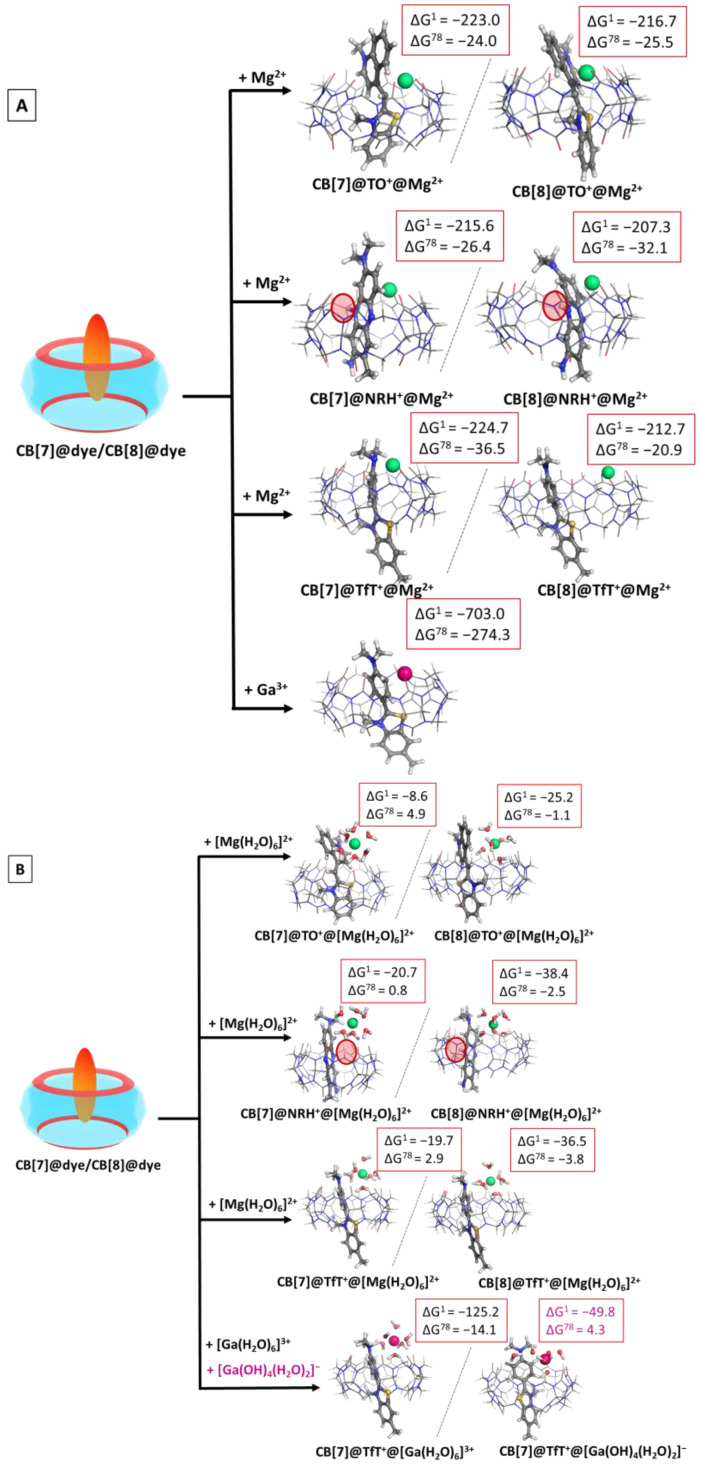
M062X/6−31G(d,p) optimized structures of the CB[7/8]@dye@metal complexes in the gas phase and the corresponding ∆G^ε^ values in kcal mol^−1^ for their formation in the gas phase (ε = 1), and water (ε = 78) at the M062X/6−31+G(d,p)//M062X/6−31G(d,p) level of theory by cooperatively binding the metal to the already formed CB[7/8]@dye complex. The results are obtained by considering the bare (**A**) and hydrated (**B**) metal cation. The hydration shell around gallium(III) is modelled as [Ga(H_2_O)_6_]^3+^/ [Ga(OH)_4_(H_2_O)_2_]^−^ in correspondence with the pK_a_ values of the metal.

Further assessment of the effect of the metal cation upon the observed host–guest interactions was carried out through modelling the cooperative association of M^n+^ to the CB@dye complexes. The obtained results in the gas phase and water solution are given in Table S5, whereas the optimized structures of the ternary complexes are depicted in Figure 5.

The calculations imply that the “bare” cation is able to form stable ternary CB@dye@M^n+^ complexes, as the observed ∆G^ε^ values are strongly negative, varying from −215.6 to −224.7 kcal mol^−1^ in the gas phase, and from −20.9 to −36.5 kcal mol^−1^ in water medium, while these values drastically decrease to −703.0 and −274.3 kcal mol^−1^ when gallium(III) is taken into account. Nonetheless, the more complex calculation achieved by modelling the hydration shell around the metal cation, takes into account additional diverse factors present in the host–guest interactions. Thus, the addition of [Mg(H_2_O)_6_]^2+^ to the initial CB[7]@dye structures appears thermodynamically impossible in aqueous solutions, as all of the obtained ∆G^78^ values are positive. It is noteworthy that this outcome stands solidly in line with the experimentally observed trends providing evidence that metal cations (divalent even stronger than monovalent) preferentially substitute guest dye molecules in CB[7], rather than cooperatively bind to the CB@dye structure, when the initial host:guest ratio is 1:1 [33]. Yet, the more voluminous cavity of CB[8] allows the cucurbituril to host a greater number of guest molecules, which is further seen in the negative Gibbs energies for the formation of ternary CB[8]@dye@M^n+^ structures. Our results suggest that in the case of CB[8], the hydrated metal cation is more likely to participate in an addition reaction to the host–dye complex rather than to substitute the dye molecule in this complex; this can be seen from the comparison of the negative ∆G^78^ values in Table S5 and the positive ones in Table S4.

Again, the effect of the hydrated gallium cation exclusively depends on the pH of the medium. The calculations imply that at pH ≈ 2, the formation of a CB[7]@TfT^+^@[Ga(H_2_O)_6_]^3+^ complex is thermodynamically possible (∆G^78^ is −14.1 kcal mol^−1^), whereas at pH ≈ 7, the corresponding CB[7]@TfT^+^@[Ga(OH)_4_(H_2_O)_2_]^−^ structure does not form spontaneously: ∆G^78^ is 4.3 kcal mol^−1^. This outcome should be attributed to the better interaction of the hexa–aqua complex with the CB[7], on one side, and to the much greater solvation in the polar aqueous medium of the ^4+^ charged former structure, as compared to the neutral latter one.

### 2.8. 2:1. CB:Dye (Denoted as 2CB@Dye Structures)

**Figure 6:**
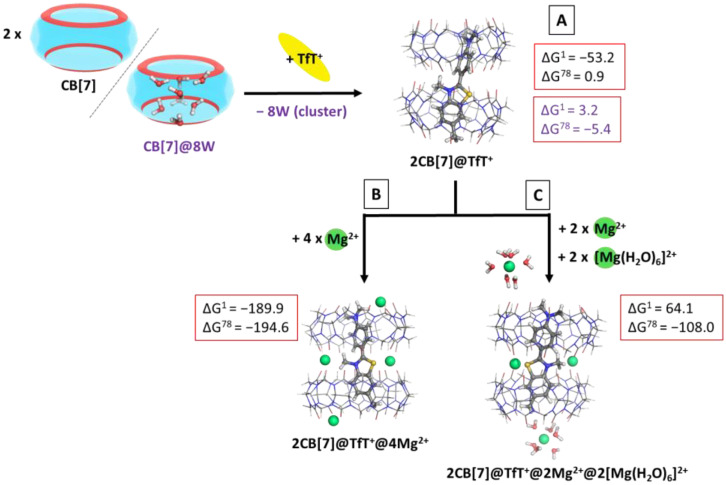
M062X/6−31G(d,p) optimized structures of the 2CB[7]@TfT^+^ (**A**), 2CB[7]@TfT^+^@4Mg^2+^ (**B**) and 2CB[7]@TfT+@2Mg^2+^@2[Mg(H_2_O)_6_]^2+^ (**C**) complexes in the gas phase and the corresponding ∆G^ε^ values in kcal mol^−1^ for their formation in the gas phase (ε = 1), and water (ε = 78) at the M062X/6−31+G(d,p)//M062X/6−31G(d,p) level of theory by cooperatively binding the metal to the already formed 2CB[7]@TfT+ complex. (**A**) The reaction is modelled by considering either CB[7] alone (results in black) or the cucurbituril with high-energy water molecules (results in purple) as an initial structure. The coming TfT^+^ fully substitutes the water cluster from the host cavity. The further addition of Mg^2+^ cations is computed by considering the bare (**B**) and hydrated (**C**) metal cations at the cavitand’s portals, whereas bare Mg^2+^ due to steric hindrance occupy the intersection between the two host molecules.

The next step in the current investigation was to model the interaction between CB[7] and the dye TfT^+^ according to reaction (R4), thus yielding a 2CB@dye structure. This outcome is observed experimentally, when the reaction of complexation is carried out in CB:dye ratio 2:1. The obtained ∆G^78^ values in a water environment are provided in the following Figure 6A, where the script (8W cluster) corresponds to a reaction modelled with a high-energy water cluster present in the initial structure of the cucurbituril, which is released in the surrounding medium upon 2CB@dye formation.

The performed calculations provide evidence of the possible complexation between CB[7] and TfT^+^ in a 2:1 ratio, as the ∆G^78^ value is very close to zero (0.9 kcal mol^−1^), which is near the error of the method. On the other hand, when taking into account the probable presence of a high-energy water cluster in the initial structure of the cucurbituril, the result decreases to −5.4 kcal mol^−1^, thus indicating the plausible host–guest interaction. These results stay firmly in line with the experimentally observed trends [33].

#### Forming Molecular Architectures

An intriguing aspect of the metal-assisted complexation of fluorogenic dyes by cucurbiturils is the effect of divalent cations on the 2CB[7]@TfT+ complex. Unexpectedly, the metal cations associate cooperatively to the structure, as experimentally observed [33], thus enhancing the fluorescent yield manifold. Therefore, we modelled the effect of magnesium cations added to the 2CB[7]@TfT^+^ complex according to reaction (R5). The obtained results are depicted in Figure 6B,C.

It is noteworthy that our theoretical results strongly correspond to the experimentally provided ones, as the addition of magnesium cations distinctively decreases the calculated Gibbs energies of complexation: −189.9/−194.6 and 64.1/−108.0 kcal mol^−1^ for the formation of 2CB[7]@TfT+@4Mg2+ and 2CB[7]@TfT^+^@2Mg^2+^@2[Mg(H_2_O)_6_] (Figure 6B,C), as compared to 0.9/−5.4 kcal mol^−1^ for the formation of 2CB[7]@TfT^+^ (Figure 6A). This indicates a strong inclination toward the formation of a highly fluorescent supramolecular nanocapsule, mainly due to the stabilization provided by the magnesium cations not only as lids at the two outer portals of the CB[7], but also at the intersection amid the host molecules. Furthermore, the arising ion–dipole interactions of attraction between the metal cations and the carbonyl groups are seen in the charge transferred in the region of the O-atoms: it is by 0.01 to 0.06e^−^ greater (Hirshfeld analysis) as compared to the metal-free structure. By sealing the space between the two CB units, the metal cations further promote the encapsulation of the guest in the host cavity (thus decreasing the influence of the polar solvent) and additionally rigidify the supramolecular nanocapsule (consequently restricting the torsional motion in the TfT^+^ ion). Hence, the fluorescent yield increases manifold.

## 3. Methods

**Figure 7:**
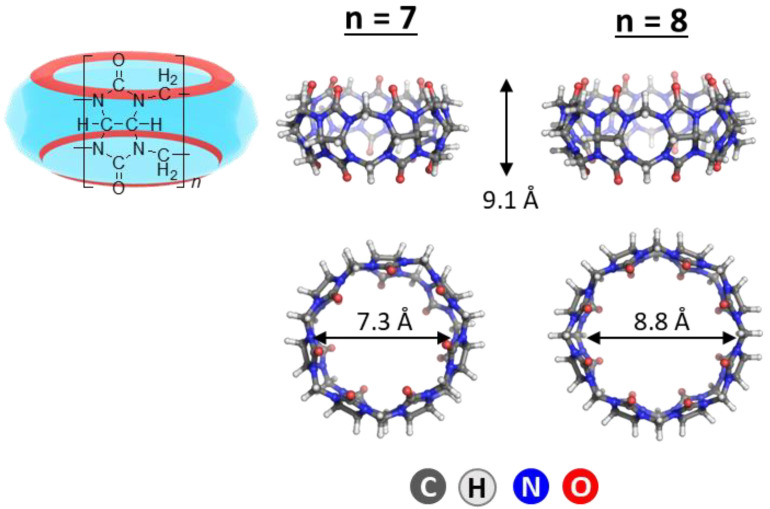
M062X/ 6−31G(d,p) optimized structures of CB[7] and CB[8]. The corresponding height and portal diameters are given in Å.

The M062X functional [56] and 6−31G(d,p) basis set were employed in optimizing the structures of the guest dye molecules, the host CB[7] and CB[8] systems and their complexes, and evaluating the respective electronic energies, E_el_. M062X functional has been shown to be efficient in main group thermochemistry, kinetics, and non-covalent interactions calculations. Furthermore, our previous studies [51,52], exploring the metal-assisted cucurbituril chemistry, as well as calculations regarding other macrocyclic structures, such as cyclodextrins [57] and calixarenes [58], proved this particular combination of method and basis set reliable when the aim is obtaining a general perspective of controlling key factors. The Gaussian 09 program [59] was employed in performing the calculations. The required initial constructs of CB[7/8] were derived from the Cambridge Crystallographic Data Centre (CCSD), namely TUHGAG for CB[7] (from Ref. [60]), and BATWEA for CB[8] (from Ref. [61]). The optimized host molecules in the gas phase along with the corresponding structural data are given in Figure 7.

In order to assess the effect of the increased sophistication of the employed basis set on the calculated energies, single point calculations (using the optimized M062X/6−31G(d,p) structures) were performed and the E_el_ at the higher M062X/6−31+G(d,p) level of theory were evaluated. Electronic energies obtained at both levels of theory (M062X/6−31G(d,p) and M062X/6−31+G(d,p)//M062X/6−31G(d,p)) were used alongside the subsequent evaluations.

Frequency calculations for each optimized structure were performed at the M062X/6−31G(d,p) level of theory. No imaginary frequency was found for the lowest energy configurations of any of the optimized structures. The vibrational frequencies were used to compute the thermal energies, E_th_, including zero-point energy, and entropies, S. The differences ΔE_el_, ΔE_th_, PΔV (work term) and ΔS between the products and reactants were used to evaluate the gas-phase free energy of the complex formation, ΔG^1^, at T = 298.15 K, according to Equation (1):

ΔG^1^ = ΔE_el_ + ΔE_th_ + PΔV–TΔS (1)

Additional single point calculations at the M062X/6−31+G(d,p)//M062X /6−31G(d,p) level in a water environment (ε = 78) using the solvation model based on density (SMD) method [62] account for the solvation effects. The solvation free energy is obtained as the difference between the SMD calculated and the gas phase energies: ∆G^78^ ≈ E_el_^78^–E_el_. The ∆G^78^ for the overall reaction is calculated as the sum of the Gibbs energy in gas phase and the resulting Gibbs energies of solvation of the products minus the solvation ∆G^78^ of the reactants, according to the formula Equation (2):

∆G^78^ = ∆G^1^ + ∆G^78^_solv_ (products)–∆G^78^_solv_ (reactants) (2)

The outcome from the thus modelled reactions is thermodynamically possible, if ∆G^78^ < 0 (negative ∆G^78^) and is thermodynamically impossible when ∆G^78^ > 0 (positive ∆G^78^).

The molecular graphics images were generated by the PyMOL [63] molecular graphics system.

## 4. Conclusions

The presented herewith theoretical results delineate physicochemical characteristics of high significance that influence the complexation abilities of the CB family with fluorogenic dyes by studying the reactions of the formation between the two representatives CB[7/8] and neutral red, thioflavin T and thiazole orange. A thorough comparison with the available experimental data in the literature has been provided, hence a clearer picture is drawn through the combination of the resources of both theory and experiment. The analysis of the obtained calculations underlines the significance of the pH region where the reactions take place: due to the arising ion–dipole interactions between the carbonyl-laced entrances of the cucurbiturils and the protonated dye, the CB[7/8] form much stronger complexes with the positively charged structure as compared to the corresponding neutral one. The presence/absence of high-energy water molecules in the inner cavity of the host has also been considered and the arising dependencies discussed. Furthermore, the cavity size of the host turns out to be of great significance, since a fine-tuned balance between the possible interactions with the guest and the “filling” of the inner cavity of the CB[n] exists. In this regard, the effect of the metal cation is two-sided: it competitively substitutes the guest dye in the smaller cavitand, whereas a cooperative addition is observed in the case of the more voluminous CB[8]. The intriguing case of the formation of the 2CB[7]@TfT+ sandwiching complex has also been modelled and the effect of metal cations upon its structure assessed. Our calculations reveal that Mg^2+^ cations strongly promote the complexation, as the energy gain increases approximately 40 times in the presence of the metal ions. Additionally, Mg^2+^ ions stabilize and rigidify the structure, which explains the manifold enhanced fluorescence yield of the supramolecular nanocapsule observed experimentally.

## Figures and Tables

**Table 1:** Names and chemical structures of the fluorogenic dyes used in the current study (without the counter ion).

Trivial Name	Abbreviation	IUPAC Name	Chemical Structure
Thioflavin T	TfT	2-[4-(Dimethylamino)phenyl]−3,6-dimethyl−1,3-benzothiazol−3-ium chloride	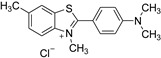
Thiazole orange	TO	1-Methyl−4-[(3-methyl−2(3H)-benzothiazolylidene)methyl]quinolinium iodide	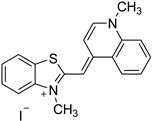
Neutral red	NR	3-amino−7-dimethylamino−2-methylphenazine hydrochloride	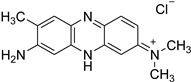

## Data Availability

The data presented in this study are available on request from the corresponding author.

## Supplementary Materials

The following supporting information can be downloaded at: https://www.mdpi.com/article/10.3390/molecules28041540/s1, Figure S1: M062X/6−31G(d,p) optimized structures of the CB[7/8] complexes with bare (non-hydrated) and hydrated metal cations in the gas phase; ∆G^ε^ values in kcal mol^−1^ for the complex formation in gas phase (ε = 1), and water (ε = 78) at the M062X/6−31+G(d,p)//M062X/6−31G(d,p) level of theory; Table S1: M062X/6−31G(d,p) calculated thermodynamic parameters in kcal mol^−1^; Table S2: Gibbs energies of CB@dye formation in a gas phase (∆G^1^) and aqueous solution (∆G^78^) in kcal mol^−1^; Table S3: Gibbs energies of CB@dye formation in a gas phase (∆G^1^) and aqueous solution (∆G^78^) in kcal mol^−1^, where the initial CB[7]@8W is considered; Table S4: Gibbs energies of CB@M^n+^ formation through substitution of the dye molecule in a gas phase (∆G^1^) and aqueous solution (∆G^78^) in kcal mol^−1^; Table S5: Gibbs energies of CB@dye@M^n+^ formation through addition of metal cation in a gas phase (∆G^1^) and aqueous solution (∆G^78^) in kcal mol^−1^.
