# Supplementary material for: Metal-Assisted Complexation of Fluorogenic Dyes by Cucurbit[7]uril and Cucurbit[8]uril: A DFT Evaluation of the Key Factors Governing the Host–Guest Recognition
Source: Molecules. 2023 Feb 5;28(4):1540. doi: 10.3390/molecules28041540 (PMC9966945; doi:10.3390/molecules28041540)
Supplement: Supplementary file 1 [file molecules-28-01540-s001.zip › molecules-2186549-supplementary.pdf]

Supplementary Materials

Metal–Assisted Complexation of Fluorogenic Dyes by Cucurbit[7]uril and Cucurbit[8]uril: A DFT Evaluation of the Key Factors Governing the Host–Guest Recognition

Nikoleta Kircheva [1], Stefan Dobrev [1], Lyubima Dasheva [2], Valya Nikolova [2], Silvia Angelova [1,*] and Todor Dudev [2,*]

[1] Institute of Optical Materials and Technologies "Acad. J. Malinowski", Bulgarian Academy of Sciences, 1113 Sofia, Bulgaria
[2] Faculty of Chemistry and Pharmacy, Sofia University "St. Kliment Ohridski", 1164 Sofia, Bulgaria
* Correspondence: sea@iomt.bas.bg (S.A.); t.dudev@chem.uni−sofia.bg (T.D.)

Table S1. M062X/6−31G(d,p) calculated thermodynamic parameters in kcal mol^{-1}.

Reaction	ΔH	TΔS	ΔG^{1}	ΔG^{78}
$CB[7] + TO^{+} \rightarrow CB[7]@TO^{+}$	−64.0	−23.6	−40.5	−12.0
$CB[7] + TfT^{+} \rightarrow CB[7]@TfT^{+}$	−69.6	−22.1	−47.5	−12.3
$CB[7] + NR^{0} \rightarrow CB[7]@NR^{0}$	−28.1	−19.5	−8.6	1.3
$CB[7] + NRH^{+} \rightarrow CB[7]@NRH^{+}$	−65.5	−18.7	−46.7	−9.4
$CB[8] + TO^{+} \rightarrow CB[8]@TO^{+}$	−62.4	−21.9	−40.5	−11.1
$CB[8] + TfT^{+} \rightarrow CB[8]@TfT^{+}$	−61.9	−21.6	−40.3	−6.7
$CB[8] + NR^{0} \rightarrow CB[8]@NR^{0}$	−29.1	−21.1	−8.0	5.0
$CB[8] + NRH^{+} \rightarrow CB[8]@NRH^{+}$	−60.3	−20.6	−39.8	−1.5
Substitution				
$CB[7]@TO^{+} + Mg^{2+} \rightarrow CB[7]@Mg^{2+} + TO^{+}$	−245.9	10.2	−256.1	−35.9
$CB[7]@TfT^{+} + Mg^{2+} \rightarrow CB[7]@Mg^{2+} + TfT^{+}$	−240.3	8.8	−249.1	−35.6
$CB[7]@NRH^{+} + Mg^{2+} \rightarrow CB[7]@Mg^{2+} + NRH^{+}$	−244.5	5.4	−249.8	−38.5
$CB[8]@TO^{+} + Mg^{2+} \rightarrow CB[8]@Mg^{2+} + TO^{+}$	−243.1	7.5	−250.6	−34.5
$CB[8]@TfT^{+} + Mg^{2+} \rightarrow CB[8]@Mg^{2+} + TfT^{+}$	−236.7	7.1	−250.8	−38.9
$CB[8]@NRH^{+} + Mg^{2+} \rightarrow CB[8]@Mg^{2+} + NRH^{+}$	−245.2	6.1	−251.3	−44.1
$CB[7]@TO^{+} + [Mg(H_2O)_6]^{2+} \rightarrow CB[7]@[Mg(H_2O)_6]^{2+} + TO^{+}$	−92.4	2.2	−94.7	−15.6
$CB[7]@Tft^{+} + [Mg(H_2O)_6]^{2+} \rightarrow CB[7]@[Mg(H_2O)_6]^{2+} + TfT^{+}$	−86.8	0.8	−87.6	−15.3
$CB[7]@NRH^{+} + [Mg(H_2O)_6]^{2+} \rightarrow CB[7]@[Mg(H_2O)_6]^{2+} + NRH^{+}$	−91.0	−2.6	−88.4	−18.2
$CB[8]@TO^{+} + [Mg(H_2O)_6]^{2+} \rightarrow CB[8]@[Mg(H_2O)_6]^{2+} + TO^{+}$	−82.4	−3.9	−78.6	4.6
$CB[8]@Tft^{+} + [Mg(H_2O)_6]^{2+} \rightarrow CB[8]@[Mg(H_2O)_6]^{2+} + TfT^{+}$	−83.0	−4.2	−78.7	0.2
$CB[8]@NRH^{+} + [Mg(H_2O)_6]^{2+} \rightarrow CB[8]@[Mg(H_2O)_6]^{2+} + NRH^{+}$	−84.5	−5.2	−79.3	−5.0
Addition				
$CB[7]@TO^{+} + Mg^{2+} \rightarrow CB[7]@TO^{+}@Mg^{2+}$	−242.4	−11.5	−230.9	−32.0
$CB[7]@TfT^{+} + Mg^{2+} \rightarrow CB[7]@TfT^{+}@Mg^{2+}$	−243.2	−10.1	−233.1	−45.9

$CB[7]@NRH^{+} + Mg^{2+} \rightarrow CB[7]@NRH^{+}@Mg^{2+}$	−236.9	−12.9	−224.1	−35.5
$CB[8]@TO^{+} + Mg^{2+} \rightarrow CB[8]@TO^{+}@Mg^{2+}$	−235.3	−10.4	−224.9	−34.1
$CB[8]@TfT^{+} + Mg^{2+} \rightarrow CB[8]@TfT^{+}@Mg^{2+}$	−229.9	−13.0	−216.9	−43.1
$CB[8]@NRH^{+} + Mg^{2+} \rightarrow CB[8]@NRH^{+}@Mg^{2+}$	−235.4	−13.4	−222.0	−31.0
$CB[7]@TO^{+} + [Mg(H_2O)_6]^{2+} \rightarrow CB[7]@TO^{+}@[Mg(H_2O)_6]^{2+}$	−28.3	−15.9	−12.4	−1.0
$CB[7]@TfT^{+} + [Mg(H_2O)_6]^{2+} \rightarrow CB[7]@TfT^{+}@[Mg(H_2O)_6]^{2+}$	−51.1	−23.9	−27.1	−7.0
$CB[7]@NRH^{+} + [Mg(H_2O)_6]^{2+} \rightarrow CB[7]@NRH^{+}@[Mg(H_2O)_6]^{2+}$	−45.5	−20.0	−25.5	−6.4
$CB[8]@TO^{+} + [Mg(H_2O)_6]^{2+} \rightarrow CB[8]@TO^{+}@[Mg(H_2O)_6]^{2+}$	−52.0	−20.3	−31.7	−10.1
$CB[8]@TfT^{+} + [Mg(H_2O)_6]^{2+} \rightarrow CB[8]@TfT^{+}@[Mg(H_2O)_6]^{2+}$	−67.8	−22.0	−45.8	−12.6
$CB[8]@NRH^{+} + [Mg(H_2O)_6]^{2+} \rightarrow CB[8]@NRH^{+}@[Mg(H_2O)_6]^{2+}$	−69.1	−24.8	−44.3	−14.1

Table S2. Gibbs energies of CB@dye formation in a gas phase (ΔG^{1}) and aqueous solution (ΔG^{78}) in kcal mol^{-1}.

Reaction	ΔG^{1}	ΔG^{78}
$CB[7] + TO^{+} \rightarrow CB[7]@TO^{+}$	−37.4	−5.0
$CB[7] + TfT^{+} \rightarrow CB[7]@TfT^{+}$	−44.7	−5.5
$CB[7] + NRH^{+} \rightarrow CB[7]@NRH^{+}$	−43.9	−3.0
$CB[7] + NR^{0} \rightarrow CB[7]@NR$	−7.0	7.5
$CB[8] + TO^{+} \rightarrow CB[8]@TO^{+}$	−38.1	−4.9
$CB[8] + TfT^{+} \rightarrow CB[8]@TfT^{+}$	−38.7	−1.4
$CB[8] + NRH^{+} \rightarrow CB[8]@NRH^{+}$	−37.7	4.6
$CB[8] + NR^{0} \rightarrow CB[8]@NR^{0}$	−6.1	11.9

Table S3. Gibbs energies of CB@dye formation in a gas phase (ΔG^{1}) and aqueous solution (ΔG^{78}) in kcal mol^{-1}, where the initial CB[7]@8W is considered.

Reaction	ΔG^{1}	ΔG^{78}
$CB[7] + TO^{+} \rightarrow CB[7]@TO^{+}$	−9.1	−8.2
$CB[7] + TfT^{+} \rightarrow CB[7]@TfT^{+}$	−16.5	−8.7
$CB[7] + NRH^{+} \rightarrow CB[7]@NRH^{+}$	−15.7	−6.2
$CB[7] + NR^{0} \rightarrow CB[7]@NR$	21.3	4.3

Table S4. Gibbs energies of CB@M^{n+} formation through substitution of the dye molecule in a gas phase (ΔG^{1}) and aqueous solution (ΔG^{78}) in kcal mol^{-1}.

Substitution	ΔG^{1}	ΔG^{78}
$CB[7]@TO^{+} + Mg^{2+} \rightarrow CB[7]@Mg^{2+} + TO^{+}$	−249.9	−33.0
$CB[7]@NRH^{+} + Mg^{2+} \rightarrow CB[7]@Mg^{2+} + NRH^{+}$	−238.0	−35.0
$CB[7]@TfT^{+} + Mg^{2+} \rightarrow CB[7]@Mg^{2+} + TfT^{+}$	−242.6	−32.5
$CB[7]@TfT^{+} + Ga^{3+} \rightarrow CB[7]@Ga^{3+} + TfT^{+}$	−704.3	−240.4
$CB[8]@TO^{+} + Mg^{2+} \rightarrow CB[8]@Mg^{2+} + TO^{+}$	−244.3	−30.6
$CB[8]@TfT^{+} + Mg^{2+} \rightarrow CB[8]@Mg^{2+} + TfT^{+}$	−243.8	−34.1
$CB[8]@NRH^{+} + Mg^{2+} \rightarrow CB[8]@Mg^{2+} + NRH^{+}$	−244.8	−40.2

$CB[7]@TO^{+} + [Mg(H_2O)_6]^{2+} \rightarrow CB[7]@[Mg(H_2O)_6]^{2+} + TO^{+}$	−88.2	−10.0
$CB[7]@NRH^{+} + [Mg(H_2O)_6]^{2+} \rightarrow CB[7]@[Mg(H_2O)_6]^{2+} + NRH^{+}$	−81.7	−12.0
$CB[7]@Tft^{+} + [Mg(H_2O)_6]^{2+} \rightarrow CB[7]@[Mg(H_2O)_6]^{2+} + TfT^{+}$	−80.9	−9.5
$CB[7]@TfT^{+} + [Ga(H_2O)_6]^{3+} \rightarrow CB[7]@[Ga(H_2O)_6]^{3+} + TfT^{+}$	−224.4	−28.3
$CB[7]@TfT^{+} + [Ga(OH)_4(H_2O)_2]^{-} \rightarrow CB[7]@[Ga(OH)_4(H_2O)_2]^{-} + TfT^{+}$	12.3	−6.5
$CB[8]@TO^{+} + [Mg(H_2O)_6]^{2+} \rightarrow CB[8]@[Mg(H_2O)_6]^{2+} + TO^{+}$	−74.3	8.5
$CB[8]@Tft^{+} + [Mg(H_2O)_6]^{2+} \rightarrow CB[8]@[Mg(H_2O)_6]^{2+} + TfT^{+}$	−73.7	5.0
$CB[8]@NRH^{+} + [Mg(H_2O)_6]^{2+} \rightarrow CB[8]@[Mg(H_2O)_6]^{2+} + NRH^{+}$	−74.7	−1.0

Table S5. Gibbs energies of CB@dye@M^{n+} formation through addition of metal cation in a gas phase (ΔG^{1}) and aqueous solution (ΔG^{78}) in kcal mol^{-1}.

Addition	ΔG^{1}	ΔG^{78}
$CB[7]@TO^{+} + Mg^{2+} \rightarrow CB[7]@TO^{+}@Mg^{2+}$	−223.0	−24.0
$CB[7]@NRH^{+} + Mg^{2+} \rightarrow CB[7]@NRH^{+}@Mg^{2+}$	−215.6	−26.4
$CB[7]@TfT^{+} + Mg^{2+} \rightarrow CB[7]@TfT^{+}@Mg^{2+}$	−224.7	−36.5
$CB[7]@TfT^{+} + Ga^{3+} \rightarrow CB[7]@TfT^{+}@Ga^{3+}$	−703.0	−274.3
$CB[8]@TO^{+} + Mg^{2+} \rightarrow CB[8]@TO^{+}@Mg^{2+}$	−216.7	−25.5
$CB[8]@TfT^{+} + Mg^{2+} \rightarrow CB[8]@TfT^{+}@Mg^{2+}$	−207.3	−32.1
$CB[8]@NRH^{+} + Mg^{2+} \rightarrow CB[8]@NRH^{+}@Mg^{2+}$	−212.7	−20.9
$CB[7]@TO^{+} + [Mg(H_2O)_6]^{2+} \rightarrow CB[7]@TO^{+}@[Mg(H_2O)_6]^{2+}$	−8.6	4.9
$CB[7]@NRH^{+} + [Mg(H_2O)_6]^{2+} \rightarrow CB[7]@NRH^{+}@[Mg(H_2O)_6]^{2+}$	−20.7	0.8
$CB[7]@TfT^{+} + [Mg(H_2O)_6]^{2+} \rightarrow CB[7]@TfT^{+}@[Mg(H_2O)_6]^{2+}$	−19.7	2.9
$CB[7]@TfT^{+} + [Ga(H_2O)_6]^{3+} \rightarrow CB[7]@TfT^{+}@[Ga(H_2O)_6]^{3+}$	−125.2	−14.1
$CB[7]@TfT^{+} + [Ga(OH)_4(H_2O)_2]^{-} \rightarrow CB[7]@TfT^{+}@[Ga(OH)_4(H_2O)_2]^{-}$	−49.8	4.3
$CB[8]@TO^{+} + [Mg(H_2O)_6]^{2+} \rightarrow CB[8]@TO^{+}@[Mg(H_2O)_6]^{2+}$	−25.2	−1.1
$CB[8]@TfT^{+} + [Mg(H_2O)_6]^{2+} \rightarrow CB[8]@TfT^{+}@[Mg(H_2O)_6]^{2+}$	−38.4	−2.5
$CB[8]@NRH^{+} + [Mg(H_2O)_6]^{2+} \rightarrow CB[8]@NRH^{+}@[Mg(H_2O)_6]^{2+}$	−36.5	−3.8

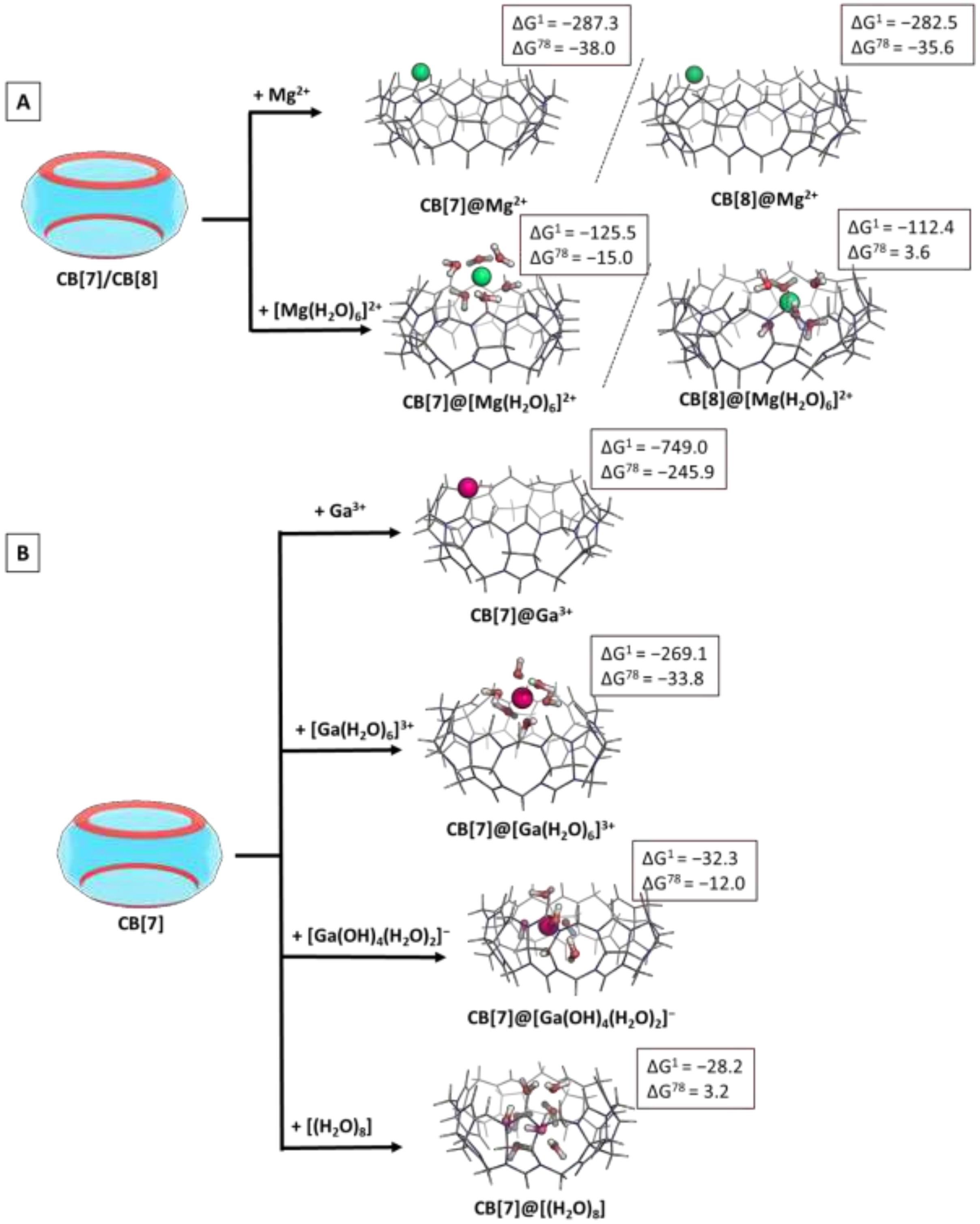

Figure S1. M062X/6−31G(d,p) optimized structures of the CB[7/8] complexes with bare (non−hydrated) and hydrated Mg^{2+} (A) cations, Ga^{3+} (B) cations and eight water molecules (B) in the gas phase; ΔG^{ε} values in kcal mol^{-1} for the complex formation in gas phase (ε = 1), and water (ε = 78) at the M062X/6−31+G(d,p)//M062X/6−31G(d,p) level of theory.
